# Dynamical Artifacts in Knitted Resistive Strain Sensors: Effects of Conductive Yarns, Knitting Structures, and Loading Rates

**DOI:** 10.3390/s26062010

**Published:** 2026-03-23

**Authors:** Alexander Oks Junior, Alexander Okss, Alexei Katashev, Uģis Briedis

**Affiliations:** 1 Institute of Architecture and Design, Riga Technical University, Kipsalas 6, LV-1048 Riga, Latvia; 2 Institute of Mechanical and Biomedical Engineering, Riga Technical University, Kipsalas 6b, LV-1048 Riga, Latvia

**Keywords:** dynamical artifacts, knitted resistive sensors, smart textiles, conductive yarns

## Abstract

This study investigates the dynamic artifacts (DAs) in knitted resistive strain sensors (KRSS) subjected to various deformation types, including stair-wise, trapezoidal, and triangle-type deformations. The presence of DAs, characterized by sharp peak-wise increases in resistance followed by a gradual decline, was observed across all KRSS samples. The amplitude of DA peaks increased with higher deformation velocities within the investigated range of 2.6–40 cm/s. The study also identified the temporal offset between resistance and deformation during linear deformation, suggesting a complex mechanism underlying DAs. The results demonstrate that DAs are most prominent in stepwise and trapezoidal deformations, while continuous deformations like triangle-type loading partially mask these artifacts. The resistance signals were recorded at a sampling rate of 150 Hz, with temporal desynchronization between recorded parameters not exceeding 6.7 ms, enabling the observation of dynamic effects. Manifestation of DAs in KRSS degrades the metrological characteristics of KRSS and cannot be ignored. This paper provides insights into the relationship between KRSS structure, deformation velocity, and DA behavior, and provides an experimental basis for future compensation approaches to mitigate the impact of DAs on measurement accuracy.

## 1. Introduction

In recent years, the development of functionalized “smart” garments has attracted significant interest due to their potential to transform everyday clothing into highly functional, high-value products. One of the most attractive directions in smart textiles is the incorporation of embedded textile strain or pressure sensors. Such devices enable the recording of human biomechanical parameters for medical, sports, or virtual reality applications. Several prototypes have already been developed and described in numerous publications [1,2,3,4,5,6,7].

Textile strain sensors could be created using various manufacturing techniques, including weaving, knitting, embroidering, printing, or a combination of these methods [2]. Typically, these sensors are made using electro-conductive yarns, such as metal-plated or impregnated yarns, or coatings, such as graphite-based materials, and they primarily function as resistive strain or pressure gauges. The advantage of these sensors is that they can be integrated into garments together with electro-conductive pathways without compromising comfort. As an example, knitting technology has gained popularity as it allows for the integration of sensors during the manufacturing process using commercially available knitting equipment.

The principle behind resistive knitted strain sensors is relatively straightforward: as the fabric deforms, the number of electrical contacts between conductive yarn loops within the knitted structure changes, leading to variations in resistance. These sensors demonstrate high sensitivity and can withstand elongations up to 20–30% [3]. Furthermore, their characteristics can be controlled by selecting the type of conductive yarn and the structure of the knitted fabric, at least in theory.

Despite such promising prospects, knitted resistive strain sensors (KRSS) face challenges due to their poor metrological characteristics: KRSS often exhibit non-linearity and hysteresis [4]. In addition to these, KRSS demonstrates a peculiar phenomenon: under dynamic loading, the resistance of KRSS displays sharp peak-wise changes at the beginning of deformation, regardless of whether it is being loaded or unloaded. This phenomenon, referred to in the text as a dynamical artifact (DA), has been observed in knitted sensors in various publications [5,6,7]. A similar phenomenon was also observed in textile-based sensors produced using printing/coating technology with various electro-conductive paint fillers, as well as in polymer film piezo resistive transducers [8,9]. Up to now, researchers have generally ignored the existence of DA or tried to address DAs by using various filtering methods, which is challenging, as it may impact KRSS sensitivity and complicate data processing.

For the first time, the existence of DA was mentioned in the literature for the case of textile polymer-coated sensors, but this phenomenon was not studied in detail [10]. To the authors’ knowledge, only one research group has attempted to study and explain DA so far [9]. They worked on the development of a piezo-resistive pressure sensor based on a polymer film with embedded conductive nano-particles and tried to explain the DA using a 2D model based on tunneling current between nano-particles and percolation theory. These researchers also proposed to use DA to increase the sensitivity of polymer film sensors for “on/off” applications instead of filtering or compensating them. Although the influence of loading rate on strain sensor signals is well known, the dynamic artifacts arising during rapid deformation are rarely analyzed systematically, and their physical origin remains insufficiently described.

The present paper represents the first attempt to provide a detailed description of DA manifestation in KRSS and explore its dependencies on deformation, loading rate, properties of conductive yarns, and structure of the knitted fabric. This is required as the first step to be made in understanding the phenomenology of DAs and creating accurate models of KRSS behavior under dynamic load. The study employs knitted sensors, previously developed by the authors and applied for human motion monitoring as an example of KRSS [11,12].

Based on this motivation, the main contributions of this study can be summarized as follows:

- Systematic experimental investigation of dynamic artifacts (DAs) in knitted resistive strain sensors under stair-wise, trapezoidal, and triangle-type deformation profiles.
- Experimental identification of a temporal offset between deformation and resistance response, indicating non-trivial DA dynamics.
- Demonstration that normalized DA peak shapes remain invariant with loading velocity, while peak amplitudes increase with a tendency towards saturation.
- Comparative analysis of the influence of conductive yarn type, knitting structure, and stitch density on DA manifestation.
- Experimental evidence that continuous deformation profiles partially mask DAs, which is important for practical sensing applications.

## 2. Materials and Methods

### 2.1. Characteristics of Yarns and Studied Knitted Structures

**Figure 1:**
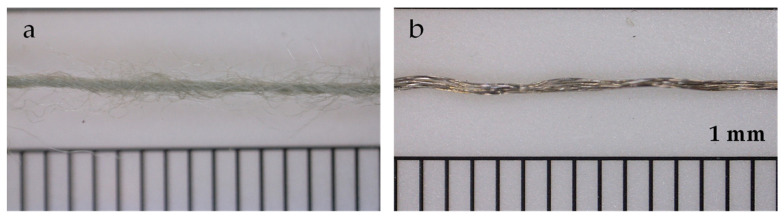
Electro conductive yarns: silver-coated polyamide (**a**), copper salt impregnated acrylic (**b**).

The structure of KRSS was knitted using combed cotton yarn (Nm 40), plated by pneumatically bound elastane/polyester yarn (22/78 dtex), and electrically conductive yarn using technology developed by the authors [3]. The two types of conductive yarn were (Figure 1). used in the study: silver-plated polyamide yarn (Shieldex^®^, Bremen, Germany) 110/34 dtex HC + B), and acrylic yarn impregnated with copper salt (Nm50, S.E. TECH, Seoul, Republic of Korea).

**Figure 2:**
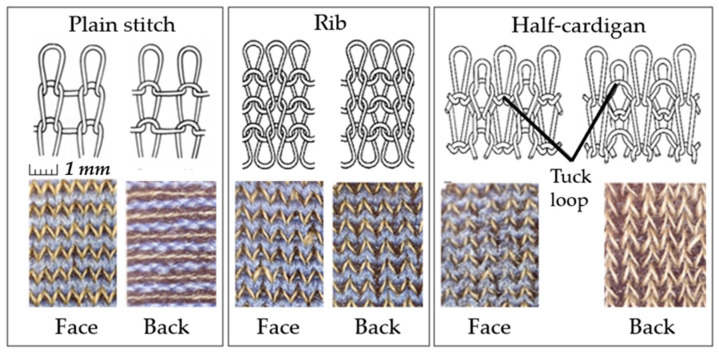
The patterns of the knitted stitches studied. (Created by authors).

Three types of stitches were studied: plain stitch (PL), rib 1 × 1 stitch (RB), and half-cardigan stitch (HC). The PL samples were manufactured with two different knitting densities, further named the low-density sample (LDS) and the high-density sample (HDS). All electroconductive fabrics were knitted using a 3¾” circular knitting machine with a 14th gauge [11]. Figure 2 shows the structures of the studied stitches. The size of the tested specimens was 155 × 40 mm.

Silver-plated polyamide (SPP) is a multifilament fiber yarn, while copper salt-impregnated acrylic (AIM) is a staple yarn. Consequently, SPP and AIM yarns exhibit significantly different hairiness, with SPP having fewer fibers sticking out and AIM having a “hairy” structure with numerous protruding fibers (Figure 1). The chosen knitting stitches also provide fabric with different properties: PL has the smoothest surface, RB is more voluminous and porous due to its double-faced structure, and HC has the highest volume and porosity due to tuck loops in its stitch [13].

### 2.2. Experimental Setup

**Figure 3:**
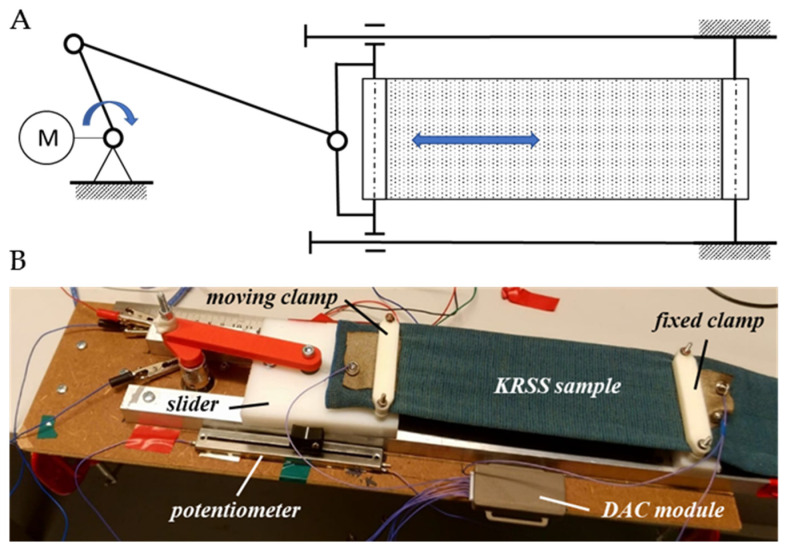
Experimental setup of KRSS cyclic deformation. (**A**) deformation principle, (**B**) deformation slider-crank device.

A custom-made slider-crank device was assembled to provide dynamic deformation of KRSS (Figure 3). Figure 3A represents the deformation principle, but Figure 3B represents the assembled deformation device. Crank and connecting rod lengths were 20 and 100 mm, respectively, which provided the slider’s motion range of 0–40 mm. Fixed clamp attached one end of the KRSS sample to the unmoving device base, while the moving clamp attached another end of the sample to the slider. The crank rotation was controlled by a step motor 17HS4401 (MotionKing Motor Industry Co., Changzhou, China) operated by an Arduino Uno circuit board. The minimal rotation step of the motor was 1.8°. This configuration allowed adjustment of KRSS deformation amplitude, velocity, and type of slider motion: stair-wise with incremental and decremental amplitudes, trapezoidal, and triangular. The slider velocity varied within a range of about 2 to 40 cm/s, which corresponds to the velocities of the deformation of the garment’s fabric in the majority of biomechanical motions.

The KRSS deformation was controlled by setting the crank’s angular position (step motor axis rotation) and measured using the linear potentiometer. The potentiometer was home-calibrated to relate changes in resistance to the position of the slider. The resistances of KRSS and the potentiometer were measured using a custom-made 8-channel data acquisition module, already exploited in the authors’ previous projects [12,14]. The module was designed on the basis of the CY8C52LP family microcontroller (model CY8C5268LTI-LP030, Infineon Technologies, Neubiberg, Germany). The module measured the voltage drop over the sensor resistance, caused by injection of the DC using the microcontroller DAC current output, thereby implementing the two-electrode-four-wire method, suggested by standard EN 16812:2016 [15]. The measured voltage was then recalculated by the module software to the resistance values in kOhms. The module enabled measurement of resistance up to 1024 kOhm; there was a possibility to set one of the 10 measurement ranges (the ranges 2, 4, 8, 16, 32, 64, 128, 256, 512, and 1024 kOhm). The sampling rate was 150 Hz; the time was measured using the internal timer of the controller. The ADC of the controller sampled the voltage over the potentiometer and the KRST resistance sequentially via an analog multiplexer. Hereby, the temporal desynchronization of the recorded time point, resistance of the potentiometer, and resistance of the KRSS sample did not exceed 6.7 milliseconds. The module communicated measured values over Bluetooth^®^ connection to the LabVIEW–based data recording software.

The uncertainties of the experimental setup were estimated as B-type uncertainty. The calculation of the uncertainty of the resistance measurement accounted for the uncertainty of the controller reference voltage, the discretization error of the 8-bit DAP, and the discretization error of the 12-bit ADC, which in total yielded a fiducial error of ±2.5%. The absolute uncertainty depended on the pre-set measurement range of the data acquisition module. The uncertainty of the time measurement was estimated to be less than maximal temporal desynchronization between measured values (see above), and was assumed equal to ±6.7 ms. The uncertainty of the extension measurement included uncertainty of the measurement of potentiometer resistance, the uncertainty of in-house calibration of the potentiometer, and hysteresis of the potentiometer, the latter provides the main contribution to the uncertainty. The resulting uncertainty of extension measurement was ±3 mm.

### 2.3. Testing Method

One specimen was prepared for each combination of stitch type, yarn type, and stitch density. Nevertheless, only plain and half-cardigan specimens were tested for all loading types: stair-wise, trapezoidal, and triangular. The rib stitch specimens and high-density plain stitch made with silver-plated yarn were tested only with triangle loading, just to demonstrate the existence of DA, because from the authors’ experience, KRSS made using this type of stitch have the shortest (in respect to other types) dynamic range and are not so useful for practical application. An overview of the studied samples and applied slider motion types is presented in Table 1. The specimens were subjected to tests of each loading type in random order.

The specimen was fixed between clamps in an unstretched state and warmed up by 10 loading–unloading cycles with maximal elongation of about 30%. After warming up, the specimen sagged because of elongation. Then the fixed clamp was released, the specimen was manually pre-stretched to remove sag, and the clamp was fastened again. If the specimen was mounted for the first time, the baseline resistance of the specimen was measured. In subsequent measurements, the specimen was manually pre-stretched in a way that its resistance was set equal to the baseline value ±5%. Hereby, the repeatability of the insertion of the sample was provided.

The stair-wise loading was provided by a gradual increment of crank angle by 9° degrees from zero to 99° in 11 steps. This provided maximal total sample elongation of about 15.0%. The loading velocity was 13.3 cm/s. At each step, the crank remained in position for 5 s.

For the trapezoidal loading, the mounted specimen was pre-stretched by initial crank positioning at 36°, which corresponded to the initial relative deformation of 2.5%. The trapezoidal deformation included stretching with a constant velocity up to pre-set relative deformation, dwell for the pre-set time, and recoil at constant velocity to the initial pre-stretched deformation of 2.5%. The parameters of the trapezoidal deformation are summarized in Table 2.

## 3. Results

### 3.1. Stair-Wise Deformation

**Figure 4:**
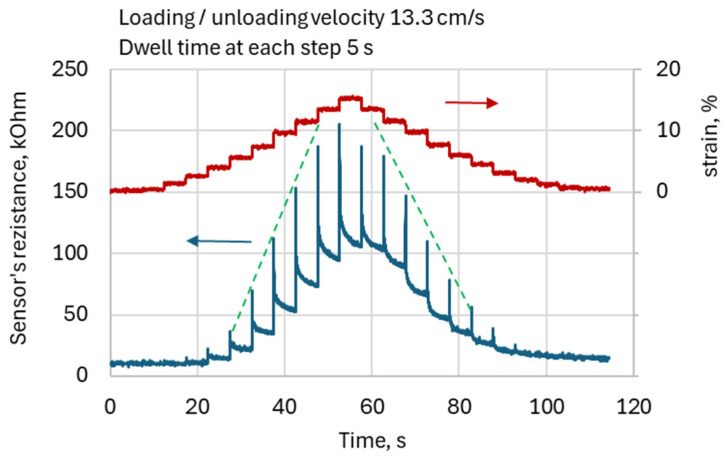
The typical waveform of the KRSS reaction (lower line) to the stepwise deformation (upper line). The dotted lines emphasize the nearly linear dependence of artifact peaks on elongation.

Figure 4 depicts the typical waveforms of KRSS resistance for the stair-wise type of slider motion. The figure illustrates KRSS response to deformation with a corresponding stepwise increase or decrease in resistance, representing the expected behavior of the sensor. However, in addition to the anticipated variations due to elongation, KRSS resistance exhibits specific DAs that manifest as sharp peak-wise increases in resistance during both extension and recoil, followed by resistance creep. As a result, the resistance waveform exhibits a remarkable “Christmas tree candles” pattern: the resistance increases sharply in a peak-like manner and then gradually declines during both extension and recoil. This characteristic pattern was observed in all tested specimens.

### 3.2. Trapezoidal Deformation

**Figure 5:**
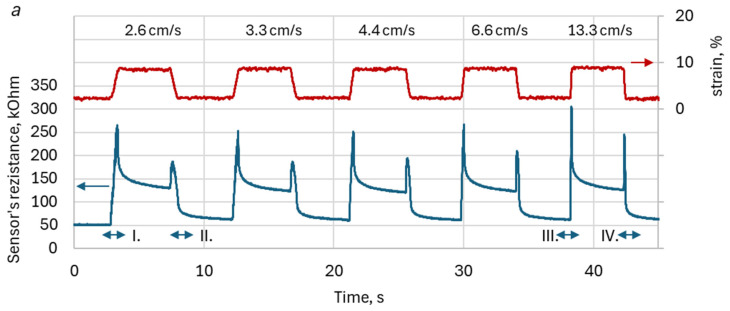

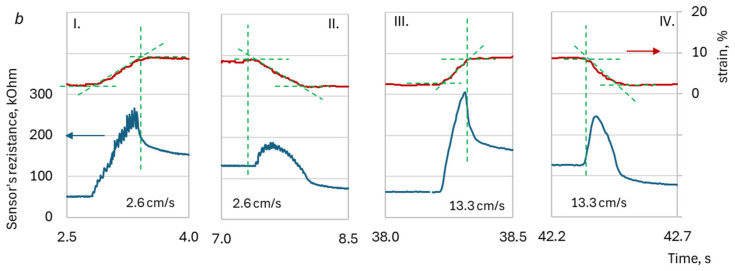
The typical sensor reaction waveform (lower lines) for the trapezoid deformation (upper lines) with different loading-unloading velocities (**a**), details of the waveforms’ edges (**b**), corresponding to low loading-unloading velocity of (zones I, II at diagram (**a**)) and high loading-unloading velocity (zones III, IV at diagram (**a**)). Data for high-density plain stitch knitted with copper impregnated yarn (PL AIM HDS).

Figure 5 presents the typical resistance-time curves for KRSS subjected to trapezoidal deformation at different slider motion velocities. The presence of DA was evident across all deformation velocities, and there was a clear tendency that the amplitude of DA peaks increased with higher deformation velocities. Analysis revealed that the DA peaks’ value is also affected by the “smoothness” of the KRSS structures. Specifically, less “fluffy” structures, such as plain stitch with high knitting density (PL PP HDS), exhibited more pronounced and sharper DAs. This demonstrates a relationship between knitting structure properties and the manifestation of DA.

Data analysis revealed several common features of DA, observed across all the KRSS samples studied:

- Resistance peak values were consistently higher during extension than during recoil, regardless of the type of KRSS sample.
- Within the range of sensor linearity, resistance peak values exhibited nearly linear dependence on KRSS deformation (green dotted lines in Figure 4) for both elongation and recoil.
- The resistance peak values, sharpness, and shape of DA peaks were found to be strongly dependent on the parameters of KRSS, such as the type of conductive yarn, knitting structure, and knitting density, as well as on the kinematic parameters of the loading-unloading cycle and deformation velocity.

The present paper, for the first time, pays attention to the most intriguing feature of DA in KRSS. To observe this feature, both deformation and sample resistance should be recorded with a proper temporal resolution that provides at least 10–20 data points for the extension or recoil stage. During linear deformation, the resistance of the KRSS sample increases to a maximum and starts to decrease before the deformation reaches the final value. In other words, the specimen is still stretched, but its resistance has already decreased. This temporal offset between resistance and deformation suggests a complex mechanism of the DA artifacts, which could not be explained by just the mechanical relaxation of the strained knitted structure. Figure 5b shows this feature for two values of KRSS deformation velocities.

One should note that resistance waveforms for low loading-unloading velocities demonstrate multiple peaks that look like ripple overlaying the smooth “principal” peak. This was caused by the vibration of the step engine motor shaft at low velocities. At high velocities, the vibration disappeared.

**Figure 6:**
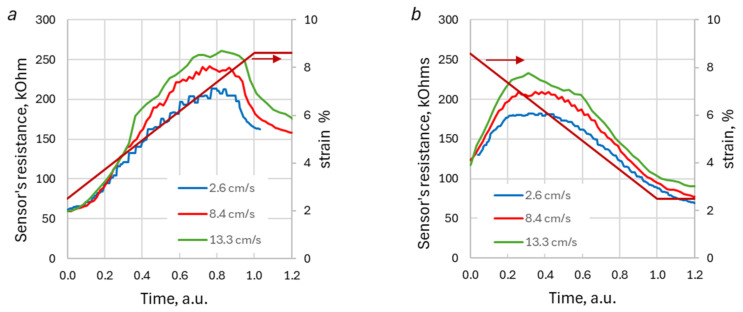

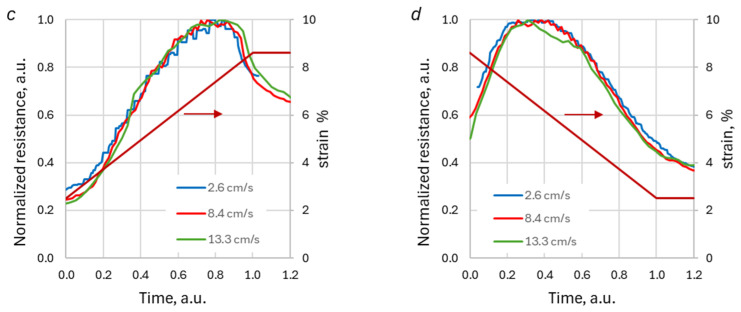
Typical sensor response for loading (**a**,**c**) and unloading (**b**,**d**) at different velocities. The timescale is normalized. The waveforms in the second row (**c**,**d**) are normalized with respect to resistance. The red line with arrow shows specimen deformation (strain). Data for the half-cardigan stitch knitted with copper impregnated yarn (PL AIM HDS).

For each type of yarn and knitting stitch, an interesting feature of the observed loading/unloading curves is the invariance of the shape of the normalized resistance peak with respect to the KRSS deformation velocity, regardless of the type of stitches, knitting densities, or yarn types. To illustrate this feature, Figure 6 demonstrates time-resistance waveforms for the HC AIM sample for three different velocities, normalized with respect to time. The waveforms in Figure 6c,d were normalized with respect to resistance, too. The time was scaled to set both loading and unloading times equal to unity. The sensor’s resistance was normalized to the peak value of the loading waveform for stretching, and to the peak value of the unloading waveform for recoil. The similarity in the peak shapes holds for both extension and recoil stages and for all studied samples.

**Figure 7:**
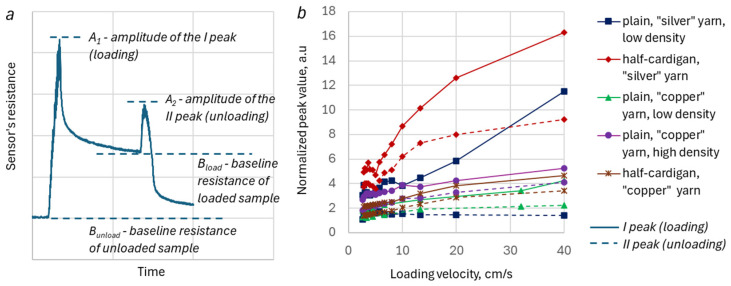
Definition of peak and baseline values (**a**). The normalized peak values vs. loading velocities (**b**).

To study the dependence of the DA peak amplitude values on the loading/unloading velocities, the peak response waveforms were normalized. Figure 7a illustrates the parameters extracted from the original waveform, corresponding to a single loading–unloading cycle. The normalized peak values were calculated as follows:

(1) $$Normalized\;peak\;value=\;\;\frac{A_{i}-B_{unload}}{B_{load}-B_{unload}}\;\;\;\;,$$

where Ai—the amplitude value of the DA peak at loading (A1) or unloading (A2),

B_load_—the baseline resistance of the loaded sample,

B_unload_—the baseline resistance of the unloaded sample.

The baseline resistance of the loaded sample is the resistance immediately before the unloading starts. The baseline resistance of the unloaded sample corresponds to the resistance of the unloaded specimen just before the loading begins. The values of the parameters B_load_ and B_unload_ for the tested samples are summarized in Table 3. The baseline values are as expected: the resistance of unloaded samples made with silver-plated yarn is in the range of ohms due to the high conductivity of the silver-plated yarn itself. Conversely, the resistance of specimens made with copper salt-impregnated yarn is in the range of tens of kiloohms. A remarkable fact is the extremely high ratio B_load_/B_unload_ for the plain silver-plated yarn sample: about 160 as opposed to 2–3 for other samples. This is an illustration of extremely high sensitivity, usually demonstrated by such a type of sensor [3].

Figure 7b shows the dependence of normalized peak values on loading/unloading velocities. All studied samples exhibited a clear increase in DA peak amplitude with a tendency to saturation for both loading and unloading cases. The possible exception is the amplitudes of the unloading peak in the plain stitch sample made with silver-plated yarn (PL SPP LDS), which demonstrated a slight decrease as unloading velocity increased; nevertheless, this variation in peak value is comparable with measurement uncertainty.

**Figure 8:**
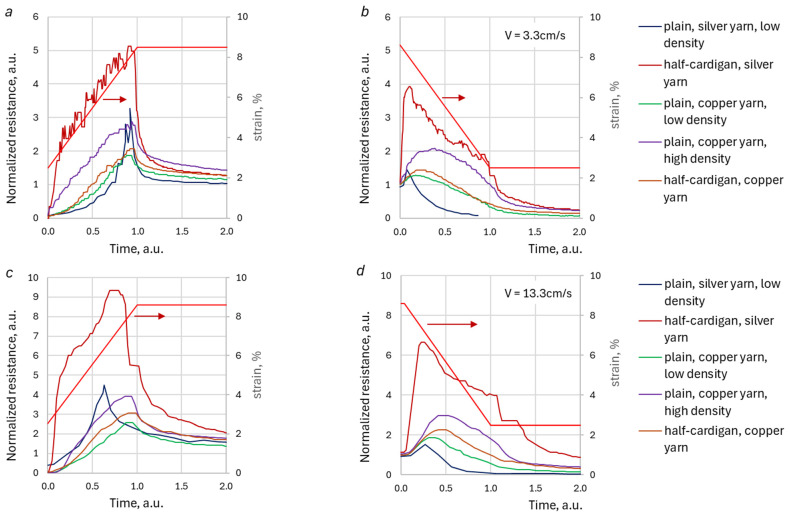
Normalized sensor reaction for different types of sensors: loading at 3.3 cm s^−1^ (**a**), unloading at 3.3 cm s^−1^ (**b**), loading at 13.3 cm s^−1^ (**c**), unloading at 13.3 cm s^−1^ (**d**). The red line with arrow shows specimen deformation (strain).

The shapes of the normalized DA peaks of the different samples for two different loading/unloading velocities are compared in Figure 8. The peaks’ waveforms are drawn versus normalized time, like one in Figure 6. These graphs allow for a comparison of the ‘sharpness’ of DAs depending on the yarn and stitch type.

### 3.3. Triangle Deformation

**Figure 9:**
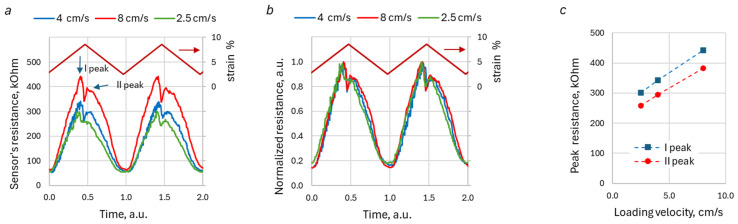
The typical sensor reaction waveform, built in a normalized time scale, for the triangle type deformation: absolute values (**a**); normalized values (**b**); peak amplitude dependence on loading velocity (**c**). The red line with arrow shows specimen deformation (strain). Data for low-density plain stitch knitted with copper impregnated yarn (PL AIM LDS).

In the case of triangle-type deformation, all the above-discussed DA features are observed, too. Figure 9 represents typical resistance waveforms for the triangle-type deformation of the PL AIM LDS sample. The time scale was normalized to the loading–unloading period, so the arbitrary time for one loading cycle was set equal to unity. Figure 9a,c clearly shows that the amplitudes of the DA peaks increase as the deformation velocity rises. At the same time, the shape of the peaks remains the same (Figure 9b), hence demonstrating invariance of loading-unloading waveform shape with respect to the deformation velocity, observed earlier for the trapezoidal type of deformation (Figure 6).

It is important to note that, for triangle-type deformation, the DAs are less distinguishable than in the case of trapezoidal deformation. DA at the start of recoil could be almost entirely masked by the decrease in resistance caused by KRSS shrinkage and the simultaneous creep of resistance, especially for lower deformation velocities.

## 4. Discussion

The previous section demonstrated the presence of DA in KRSS with different structural compositions and material characteristics. The analysis indicates that these DAs are significantly influenced by the properties of the KRSS, as well as the nature of the deformation process. The results suggest that DAs are more pronounced in less fluffed and smoother knitted structures and yarns. For instance, the amplitudes of the DA peak at loading are, generally, higher for KRSSs that contain smooth silver-plated polyamide (PL) yarn (Figure 7b). Plain stitch sample, made with this type of yarn (PL SPP LDS), exhibits sharper resistance peaks, as compared to copper salt-impregnated acrylic (AIM) yarn, which has a “hairy” structure (see Figure 8). The visually perceived increase in sharpness of the DA peak with the increase in loading/unloading velocities, which could be seen in Figure 5, is just because the waveform “shrinks” at higher velocities. The shape of the peak remains the same for all velocities (Figure 6c,d and Figure 9b), but the amplitude of the peak in general gradually increases with the increase in the loading–unloading velocity (Figure 7b and Figure 9c). Both these features are important for the understanding of the DA mechanisms, as well as the development and verification of KRSS models.

It should also be noted that the general increase in resistance with increasing deformation observed in Figure 4 is consistent with the behavior of many textile and composite piezoresistive strain sensors. Under tensile deformation, the conductive network formed by contacts between conductive yarn filaments becomes progressively disrupted, leading to a reduction in the number of effective electrical pathways and consequently to an increase in the overall resistance of the sensing structure. Similar behavior has been reported for various piezoresistive textile and polymer-based strain sensors operating through percolation-type conductive networks [16].

DAs are more prominent during processes when rapid deformation of the KRSS is accompanied by stops in stationary positions, like during stepwise or trapezoidal stretching/recoiling deformations. Such deformations are commonly encountered in various scenarios, including rehabilitation exercises involving body part movement with intermittent position fixation. In contrast, DAs can be masked in processes with continuous deformation, following, e.g., triangle or harmonic law, which are typical for applications like breathing monitoring. However, the masking effect does not imply the absence of DAs, and ignoring the impact of these artifacts can lead to inaccurate data.

It is important to note that the behavior of the PL SPP LDS stitch sometimes deviates from the described general tendencies in DA behavior. For instance, experimental data demonstrated a decrease in the second peak resistance value of this sample with an increase in unloading velocity during trapezoidal stretching/recoil tests (Figure 7b). Additionally, although the relative peak resistance values of this sample were higher than those of samples knitted with copper salt-impregnated yarn under loading conditions, they were still lower than the peak values observed in the ‘fluffier’ half-cardigan stitch with silver-plated yarn (HC SPP sample, Figure 8a,c). The authors hypothesize that these ‘contradictions’ may appear because some peak data had been missing, because of the very sharp and rapid manifestation of DAs in the PL SPP LDS stitch sample. Alongside, this sample demonstrated a low baseline resistance value (several ohms). Both these KRSS characteristics–either sampling rate or sensitivity threshold–were near the operating range limit of the data acquisition system.

Our results generally are aligned with previous research, where distinct resistance peaks were observed in KRSS knitted in a plain stitch [5,6] and in various types of double-bed knitted structures (such as double face, double left side, and interlock) [16], all fabricated using silver-plated polyamide yarns. Resistance peaks were also evident in sensors knitted in plain stitch using a qualitatively different type of conductive yarn, such as stainless steel-polyester blended staple yarn (BEKAERT BEKINTEX^®^ BK) [17]. This confirms that the presence of DAs is a fundamental property of KRSS that should be considered in the design of KRSS-based measurement and monitoring systems.

On the other hand, some studies did not demonstrate DAs in knitted sensors. For example, Atalay et al. [18,19] reported smooth resistance responses in KRSS under periodic triangular loading. The KRSS was fabricated using a compressed interlock structure as the base with a single row of silver-plated polyamide yarn, knitted in the base structure as the sensing element. This research was focused on the monitoring of breathing, so the KRSS tests were conducted at a low loading speed (0.2 cm/s). It could be inferred that, in this case, the DAs were likely very weak and may have been completely obscured by the gradual resistance change under the applied load.

**Figure 10:**
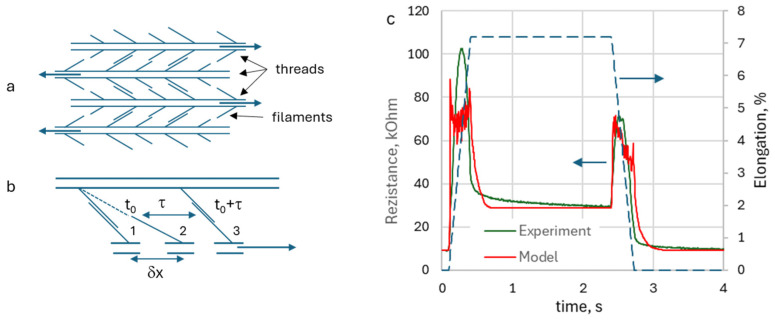
Illustration of the filament contact model structure (**a**), with demonstration of filament in contact ((**b**), position 1), disconnect of the filaments due to deformation δx ((**b**), position 2), and reconnection after relaxation time τ ((**b**), position 3), simulation results, compared with experimental data (**c**). The dashed line with arrow shows specimen deformation (elongation).

To explain the observed behavior of the knitted sensors, especially the observation of the DA, expressed as resistance peaks with a following decrease while the specimen deformation continues to increase, one could propose the following model. The conductivity of the KRSS is provided due to contacts between the side filaments of the conducting threads (Figure 10a). At deformation, the threads come into relative motion; as a result, the filaments in contact slide with respect to each other (Figure 10b, position 1), and when the local deformation reaches value δx, they disconnect (Figure 10b, position 2). The value δx is individual for each filament and depends on its length. The filament remains disconnected for a while and reconnects with another filament after some characteristic time τ (Figure 10b, position 3). This re-connection of the filaments leads to a decrease in KRSS resistance even if the deformation continues to increase.

The proposed mechanism was simulated using a simple model, composed of 1000 separate filaments, that had δx randomly uniformly distributed within the range from 0% to 1% (measured as relative deformation), and re-connection times τ randomly uniformly distributed within the range from 0 to 0.5 s. Figure 10c shows that the model gives a result that quantitatively describes the behavior of the observed DA. Future work is required to elaborate the model to achieve quantitative accuracy, too.

An intriguing observation is that similar DAs are observed in piezo resistive polymer structures: a remarkable “Christmas tree candles” pattern of sensor resistance was obtained for stepwise compression/extension of piezo resistive polymer film sensors [9]. These polymer sensors exhibit DA artifacts of varying intensity depending on the characteristics of the binder material. Less viscous binders or more elastic matrices tend to result in weaker DAs, while harder polymer matrices show more pronounced DA manifestations [8]. Several studies on the dynamic behavior of polymer piezo resistive materials have demonstrated that DAs occur independently of the filler material used, whether it is carbon black (CB), carbon nanotubes (CNT), or graphene [20,21,22,23,24,25]. A strong dependence of DA manifestation on the type of loading, strain magnitude, and loading velocity has also been observed. Additionally, a DA masking effect was noted in cases of triangle-wise or sine-wise continuous deformation: the manifestation of DA could be confused with the increase in the sensor’s resistance due to deformation [20].

The parallel between the properties of DA in polymer piezo-resistive sensors and KRSS suggests some analogy between the parameters of the underlying processes. In particular, the viscosity of the substrate material in polymer sensors corresponds to the fluffiness of KRSS, and the irregularity of filler shape in polymer sensors aligns with the hairiness of conductive yarn, filler fraction with stitch density. This similarity becomes more apparent when considering that both KRSS and piezo resistive polymers are multifractional heterogeneous piezo resistive structures. Because of such similarities, one could hypothesize that KRSS, under certain assumptions, can serve as a macroscopic electro-mechanical model for polymer piezo-resistive sensors with micro/nano conductive fillers.

The presence of DAs in KRSS introduces both challenges and opportunities. While DA potentially degrades the metrological characteristics of KRSS, compensation methods could be developed to increase the accuracy of measurements using the approach employed for the polymer piezo resistive sensor applications [26,27]. These compensation methods require a deep analysis of KRSS properties under dynamic load and the development of a dynamic model of KRSS for use in compensation procedures.

Another proposed approach involves the use of DAs to increase the sensitivity of piezo-resistive polymer sensors [9]. This approach has already been applied to KRSS, too, in an application for the monitoring of rehabilitation exercises to detect undesirable body movements [12].

Future research should include the development of mathematical models to predict the behavior of KRSS under dynamic loads and explore the potential use of DAs in measurements. Additionally, the potential similarity (in the spirit of similarity theory) in the mathematical description of the DA in KRSS and polymer piezo resistive structures should be studied. These endeavors will facilitate further understanding of DA in KRSS and its applications in smart textiles and sensor technology.

## 5. Conclusions

In summary, this study highlights the dynamic artifact (DA) behavior in knitted resistive strain sensors (KRSS) subjected to various types of deformation. The presence of DAs in KRSS degrades the metrological characteristics of KRSS and cannot be ignored. The present study demonstrates that DA peak amplitudes increase with the rise in deformation velocity and are sensitive to structural characteristics of the KRSS, including yarn composition, knitting density, and stitch type. Specifically, less “fluffy” structures, such as dense plain stitches made with silver-plated yarn, exhibit sharper and more pronounced DAs compared to those made with copper salt-impregnated yarn, indicating a direct correlation between structural smoothness and DA peak sharpness. The DAs are most prominent in stepwise and trapezoidal deformations, while they were partially masked in continuous triangular deformations.

The similarities in DA patterns between KRSS and piezo resistive polymer structures suggest potential for KRSS as a macroscopic model for polymer-based sensors, given the analogous influence of structural properties on DA manifestations. Future work should focus on developing dynamic models of KRSS that can predict sensor responses to predefined deformations and explore the potential of these models for real-time DA compensation in measurements.

## Figures and Tables

**Table 1:** Studied samples and the type of deformation.

No.	Stitch Type	Yarn	Stitch Density *	Loading Type	Abbreviation
1	Plain	Copper salt impregnated	High 109 × 162	Stair-wise/ Trapezoidal/Triangle	PL AIM HDS
2	Plain	Copper salt impregnated	Low 101 × 124	Stair-wise/Trapezoidal/Triangle	PL AIM LDS
3	Plain	Silver plated	High 120 × 150	Triangle	PL SPP HDS
4	Plain	Silver plated	Low 100 × 125	Stair-wise/Trapezoidal/Triangle	PL SPP LDS
5	Half-cardigan	Copper salt impregnated	Low 68 × 74	Stair-wise/Trapezoidal/Triangle	HC AIM
6	Half-cardigan	Silver plated	Low 68 × 74	Stair-wise/Trapezoidal/Triangle	HC SPP
7	Rib 1 × 1	Copper salt impregnated	High 71 × 134	Triangle	RB AIM HDS
8	Rib 1 × 1	Copper salt impregnated	Low 70 × 90	Triangle	RB AIM LDS

* Stitch density is given in courses × wales/10 cm.

**Table 2:** Parameters of trapezoidal deformation.

No.	Parameter	Values
1	Loading velocity, cm/s	2.6; 2.9; 3.1; 3.3; 3.6; 4.0; 4.4; 5.0; 5.7; 6.7; 8.0; 10.0; 13.3; 20.0; 32.0; 40.0
2	Relative deformation	9%; 13%; 17%
3	Dwell time, s	2; 4

To provide triangle loading, the same specimen set-up procedure was used. The loading/unloading velocities were 2.6, 4.2, and 8.4 cm/s.

**Table 3:** Baseline resistance values.

Specimens		B_load_, kOhm value (SD)	B_unload_, kOhm Value (SD)
PL AIM HDS	(plain, “copper” yarn, high density)	123 (3)	61.1 (1.0)
PL AIM LDS	(plain, “copper” yarn, low density)	39 (3)	13.0 (1.0)
PL PP LDS	(plain, “silver” yarn, low density)	0.50 (0.06)	0.0030 (0.0004)
HC AIM	(half-cardigan, “copper” yarn)	99 (6)	44.7 (1.1)
HC PP	(half-cardigan, “silver” yarn)	0.033 (0.003)	0.018 (0.002)

## Data Availability

The original contributions presented in this study are included in the article. Further inquiries can be directed to the corresponding author.

## Abbreviations

The following abbreviations are used in this manuscript:

DAs	dynamic artifacts
KRSS	knitted resistive strain sensors
PL	plain stitch
RB	rib 1 × 1 stitch
HC	half-cardigan stitch
LDS	low-density sample
HDS	high-density sample
SPP	silver-plated polyamide
AIM	copper salt-impregnated acrylic
